# A methodology to globally assess ectodomain shedding using soluble fractions from the mouse brain

**DOI:** 10.3389/fpsyt.2024.1367526

**Published:** 2024-06-19

**Authors:** Miguel Lobete, Tamel Salinas, Sara Izquierdo-Bermejo, Silvia Socas, María Jesús Oset-Gasque, M. Dolores Martín-de-Saavedra

**Affiliations:** Department of Biochemistry and Molecular Biology, School of Pharmacy, Instituto Universitario de Investigación en Neuroquímica, Universidad Complutense de Madrid, Madrid, Spain

**Keywords:** ectodomain shedding, central nervous system, mass spectrometry, sheddome, neuropsychiatric disorders, biomarkers, pharmacological targets

## Abstract

Ectodomain shedding (ES) is a fundamental process involving the proteolytic cleavage of membrane-bound proteins, leading to the release of soluble extracellular fragments (shed ectodomains) with potential paracrine and autocrine signaling functions. In the central nervous system (CNS), ES plays pivotal roles in brain development, axonal regulation, synapse formation, and disease pathogenesis, spanning from cancer to Alzheimer’s disease. Recent evidence also suggests its potential involvement in neurodevelopmental conditions like autism and schizophrenia. Past investigations of ES in the CNS have primarily relied on cell culture supernatants or cerebrospinal fluid (CSF) samples, but these methods have limitations, offering limited insights into how ES is modulated in the intact brain parenchyma. In this study, we introduce a methodology for analyzing shed ectodomains globally within rodent brain samples. Through biochemical tissue subcellular separation, mass spectrometry, and bioinformatic analysis, we show that the brain’s soluble fraction sheddome shares significant molecular and functional similarities with *in vitro* neuronal and CSF sheddomes. This approach provides a promising means of exploring ES dynamics in the CNS, allowing for the evaluation of ES at different developmental stages and pathophysiological states. This methodology has the potential to help us deepen our understanding of ES and its role in CNS function and pathology, offering new insights and opportunities for research in this field.

## 1 Introduction

Ectodomain shedding (ES) is a crucial biological process involving the cleavage of membrane-bound proteins, releasing soluble extracellular fragments known as shed ectodomains (for a review, see (1)). ES is known to regulate transmembrane proteins (canonical ES), but it has also been shown that glycosylphosphatidylinositol (GPI)-anchored proteins are regulated by this process, leading to the release of soluble proteins (2, 3). In the central nervous system (CNS), ES plays pivotal roles in brain development (4), axonal regulation (4, 5), synapse formation and transmission (6–8), and the pathogenesis of conditions ranging from cancer (9) to Alzheimer’s disease (10, 11). Recent evidence also hints at its involvement in neurodevelopmental conditions like autism and schizophrenia (12). Interestingly, ES does not necessarily lead to the end of the function of a given protein, as the shed ectodomains possess biological activity and are known to exert both auto and paracrine effects (13). Shed ectodomains play key roles in essential neuronal processes, such as synaptic maturation (6), axonal development (14, 15), neurite outgrowth (15), myelination (16), synaptic transmission (15), and neuronal synchrony (12), among other functions. Hence, investigating the brain sheddome is a fundamental approach to enhance our understanding of the molecular mechanisms contributing to brain health and disease.

Previous approaches to assess the molecular and functional composition of the brain sheddome have primarily involved collecting cell culture media (12, 17) or the analysis of cerebrospinal fluid (CSF) (12, 17, 18). Using cell culture media enables precise manipulation of experimental conditions. The analysis of the human CSF offers several advantages, including its diagnostic potential and the possibility of extracting CSF from the same individual over time. This allows for the study of the impact of therapies or time on the sheddome’s composition. However, both of these methods present inherent limitations. *In vitro* studies have well-known limitations inherent in research performed in culture, such as the inability to accurately represent disease and developmental mechanisms, along with the oversimplification of neuroinflammatory responses. On the other hand, CSF analysis relies on shed proteins capable of diffusing out of the brain parenchyma. This approach is also limited by the challenge of extracting the biological fluid in younger animals due to size constraints, and the limited CSF volume obtained from each animal poses difficulties in employing multiple analytical techniques on the same sample. Consequently, there is a crucial need in the field to identify an alternative methodology that can circumvent the limitations of the two aforementioned techniques in studying the brain sheddome. An alternative to the *in vitro* and CSF sheddome analysis could be the extraction of brain soluble fractions. This approach offers several advantages, including the use of *in vivo* models that enable the study of several aspects of development, disease, and neuroinflammation globally. Unlike CSF analysis, this method is not limited to diffusible proteins. Additionally, the amount of sample allows for versatile analysis of the same specimen using mass spectrometry, Western blot, or ELISA, to name a few.

Here, we describe, optimize, and validate a methodology for the global analysis of brain sheddomes by mass spectrometry, overcoming limitations inherent in existing approaches. Our protocol involves the dissection of mouse brain cortices, followed by mechanical tissue homogenization in a detergent-free buffer to avoid membrane solubilization. A subsequent centrifugation removes cellular debris, nuclei, and larger organelles. The supernatants are then ultracentrifuged to separate insoluble cell membranes from soluble fractions. The extracted soluble fractions contain shed ectodomains, which were initially attached to the membrane by either a transmembrane domain or a GPI anchor and were released by the proteolytic activity of sheddases. Then, liquid chromatography coupled to mass spectrometry is employed to analyze brain soluble samples. To identify proteins that could have undergone ES, known as the brain soluble fraction sheddome, we bioinformatically filter out proteins that possess a transmembrane domain or a GPI anchor according to UniProt.

Our data demonstrate the efficacy of this methodology in detecting shed ectodomains. We optimized the ultracentrifugation step, finding that 2 h at 100,000 *g* is ideal for separating membranes from soluble fractions, thereby eliminating potential contaminants. The molecular and functional composition of the brain soluble fraction sheddome, analyzed by bioinformatic tools, was strikingly similar to that of previously described sheddomes. ES of a few candidates was corroborated by Western blotting, further validating our methodology. This protocol offers a validated means of investigating sheddomes in *in vivo* experimental samples, promising insights into the impact of ES on protein function, brain physiology and its contribution to neuropsychiatric disorders.

## 2 Materials and methods

### 2.1 Animals

All animals were housed in a room with controlled photoperiod (08:00–20:00 light) and temperature (22 ± 1°C) with free access to standard food and water. Experiments were conducted according to local and European rules (directive 2010/63/EU) and were approved by the Ethical Committee of Universidad Complutense de Madrid (ref. PROEX 305.6/22). All the mice used in this study were 10-week-old C57BL6J female mice.

### 2.2 Antibodies and chemicals

The following antibodies were purchased: Primary antibodies: N-cadherin (mouse monoclonal, BD Biosciences, Cat# 610921, RRID: AB_398236), phospho-p44/42 MAPK (rabbit polyclonal, Cell Signaling Technology, Cat# 9102, RRID: AB_330744), Synaptotagmin 1 (rabbit polyclonal, Synaptic Systems, Cat# 105 103, RRID: AB_11042457), CD81 (rabbit monoclonal, Cell Signaling Technology, Cat# 10037, RRID: AB_2714207), CNTNAP2 (N-terminal, mouse monoclonal, Neuromab, Cat# 75–075, RRID: AB_2245198), CNTNAP2 (C-terminal, rabbit polyclonal, Millipore, Cat# AB5886, RRID: AB_92118), Neuroligin 1 (N-terminal, rabbit polyclonal, Alomone, Cat# ANR-035, RRID: AB_2341006), Neuroligin 3 (C-terminal, mouse monoclonal, Synaptic Systems, Cat# 129321, RRID: AB_2924997), and Neuropilin 2 (goat polyclonal, R&D Systems, Cat# AF2215, RRID: AB_2155371). Secondary antibodies: goat polyclonal anti-mouse IgG (Sigma-Aldrich, Cat# A4416, RRID: AB_258167), goat polyclonal anti-rabbit IgG (Thermo Fisher, Cat# 31460, RRID: AB_228341), and rabbit polyclonal anti-goat IgG (R&D Systems, Cat# HAF017, RRID: AB_562588).

The following reagents were employed in this study: tris (Sigma-Aldrich, Cat# 10708976001), NaCl (Panreac, Cat# 211659.1214), protease inhibitor cocktail (Thermo Fisher, formerly Roche Diagnostics, Cat# 11873580001), sodium deoxycholate (Sigma-Aldrich, Cat# D6750), sodium dodecyl sulfate (SDS; Sigma-Aldrich, Cat# L4390), Triton X-100 (Merck, Cat# T8787), iST KIT (Preomics, Cat# P.O.00001), acrylamide:bis-acrylamide (Thermo Fisher Scientific, Cat# J60126.AP), BCA (Thermo Fisher, Cat# 23225), Laemmli 2× (Bio-Rad, Cat# 1610737), β-mercaptoethanol (Sigma-Aldrich, Cat# M6250), bovine serum albumin (Sigma-Aldrich, Cat# A9647), tween20 (Sigma-Aldrich, Cat# P1379), KCl (Merck, Cat# 1.04936), and methanol (Panreac, Cat# 141091.1211).

### 2.3 Separation of membrane and soluble fractions

Ten-week-old wild-type C57BL6J female mice were sacrificed by cervical dislocation and dissected to obtain the cerebral cortices. Cortices were mechanically homogenized with a glass homogenizer in tris-based buffer (TS) containing 50 mM tris-HCl, 150 mM NaCl, and protease inhibitor cocktail (Thermo Fisher, formerly Roche Diagnostics). The total homogenate (HT) was centrifuged at 1,500 *g* for 10 min at 4°C to remove cell debris, nuclei, and other large organelles released after mechanical homogenization. The supernatant (S1) was then ultracentrifuged at 100,000 *g* (100K *g*) or 200,000 g (200K *g*) for either 1 h or 2 h at 4°C with a 100 Ti rotor (Beckman Coulter) using a Beckman XL-90 ultracentrifuge to separate the membranous from the soluble fractions. The supernatants (S2) and the precipitates (P2) were collected. P2 fractions were resuspended in TS buffer with 1% Triton X-100, 0.5% sodium deoxycholate, and 1% SDS. S1 and S2 fractions were analyzed by liquid chromatography with tandem mass spectrometry for the molecular and functional assessment of the brain soluble fraction sheddomes. HT, S1, S2 (100K *g* and 200K *g*), and P2 (100K *g* and 200K *g*) fractions were analyzed by Western blot as well.

### 2.4 Liquid chromatography with tandem mass spectrometry

Samples analysed by liquid chromatography with tandem mass spectrometry (LC-MS/MS) were prepared using the PreOmics iST kit, according to the manufacturer’s directions. Briefly, the samples were diluted with cold acetone, and, after centrifugation, the supernatant was removed. Each precipitated sample was then treated with Lyse buffer at 95°C for 10 min, transferred to a column, and the Digest solution was added. After a 2h incubation at 37°C, Stop buffer was added, followed by centrifugation for 2 min at 3,800 *g*. The resulting digest underwent washing steps with Wash 1 and Wash 2 buffers and was eluted twice with Elute buffer. The sample was subsequently dried in a Speed-Vac (Thermo-Savant) and reconstituted in LC-load. Finally, peptide concentration was determined using the Qubit system (Thermo Fisher) and calculations were performed to inject 0.5 µg into the nanoHPLC (Thermo Fisher).

For the fractionation of the S2 1 h 100K *g* sample, we used Bio-Rad’s mini-protean system, with gels consisting of a 10% acrylamide spacer gel and a 4% concentrator gel. Subsequently, 20 µg of protein boiled 5 min in sample buffer (6 mM Tris, 2% SDS, 10% glycerol, 0.5 M β-mercaptoethanol, and traces of bromophenol blue) was loaded into the wells and electrophoresis was ran at 100 V. Colloidal Coomassie (G-250) was used to stain the proteins after fixating (in 50% methanol, 2% phosphoric acid) and equilibrating (in methanol 33%, ammonium sulfate 17%, phosphoric acid 3%) the gels. The excess Coomassie was removed by performing multiple washes with milli-Q water followed by two washes with acetonitrile (ACN) alternated with rehydration of the gels with 25 mM ammonium bicarbonate (AMBI). Disulfide bridges were reduced with 10 mM DTT in 25 mM AMBI at 56°C for 30 min and blocked with 22.5 mM iodoacetamide in 25 mM AMBI for 10 min in the dark. After removal of reagent residues with two ACN washes, the gels were completely dehydrated in a Speed-Vac for 30 min, after which trypsin (recombinant proteomics grade, Thermo Fisher) was added at a ratio of 1 µg per sample tube and allowed to act overnight at 37°C. Peptides were collected in the digestion supernatant and extracted by blotting the gels with ACN. The resulting liquid was dried in the Speed-Vac and reconstituted in ACN 2% and formic acid 0.1%. Peptide concentration was measured by fluorimetry on the Thermo Fisher Qubit 3.0 system.

From the digested peptide mixture, 0.5 µg was injected into the Easy-nLC 1000 nano-HPLC, concentrated on a PEPMAP100 C18 NanoViper Trap precolumn (Thermo Fisher) and separated on a 50cm PEPMAP RSLC C18 column (Thermo Fisher) using a 2% to 40% ACN and 0.1% formic acid gradient over 120 min.

The chromatographically separated peptides were electrospray ionized in positive mode and analyzed on a Q Exactive HF mass spectrometer (Thermo Fisher) in data-dependent acquisition (DDA) mode. MS scans between 350 and 1,700 Da were performed, followed by the selection of the 10 most intense precursors (with charges between 2+ and 6+) for high collision energy dissociation (HCD) fragmentation and the acquisition of the corresponding MS/MS spectra.

Data obtained from the shotgun analysis were processed using the Proteome Discoverer software (Thermo Fisher). Peptide-spectrum matches (PSMs) of each MS/MS spectrum were identified by comparing them with theoretical mass lists from the mouse protein database in the UniProt sequence repository, using the Sequest search engine. Peptides were then assigned to their corresponding proteins utilizing the principle of parsimony to generate a “Master” protein when a peptide could be associated with multiple proteins. The percolator algorithm was used to estimate the false-positive rate (FDR) and filtered by a *q*-value <0.01 for proteins identified with high confidence.

### 2.5 Biological process enrichment analysis

The gene annotation enrichment analysis tool DAVID v6.8 was used to perform gene ontology (GO) term enrichment of biological processes (19). To determine the subset of proteins that undergo ES, we overlapped the datasets with proteins that are cell membrane bound (i.e., contain at least one transmembrane domain or GPI anchor) based on UniProt annotations (**Supplementary Table 1**). To avoid nomenclature discrepancies, all IDs were translated to entrez gene IDs. GO analysis of soluble fraction sheddome was corrected for all membrane-anchored proteins to eliminate the possibility of biasing the data toward that group of proteins. For P2 analysis, we used proteins expressed in the mouse cortex (list obtained from Human Protein Atlas). The -log_10_ of the Benjamini-corrected *p*-values were calculated and plotted for the first non-redundant categories of GOTERM_BP_DIRECT, where statistical significance is reached at a value of 1.3.

### 2.6 Protein–protein interaction network analysis

Protein–protein interaction (PPI) analysis was conducted using STRING v12.0. Network edges represent PPI confidence, using experiments and databases as the active interaction sources, with medium confidence. The network was adjusted using the k means clustering method. This approach was employed to provide a comprehensive and intuitive understanding of the functional properties of the proteins within the samples.

### 2.7 Western blot

After determining the protein concentration of each sample using the bicinchoninic acid method (Pierce BCA protein assay kit) (Thermo Fisher), the appropriate sample volume required to load an equivalent amount of protein onto the electrophoresis gels was calculated. Subsequently, Laemmli 2× buffer (Bio-Rad) and β-mercaptoethanol (Sigma-Aldrich) were added, and the prepared samples were boiled at 95°C for 2 min in a thermoblock. The samples were then loaded onto gels with varying percentages of polyacrylamide. Following electrophoretic separation at 120 V, the proteins were transferred to polyvinylidene fluoride (PVDF) membranes activated in methanol. Once transferred, they were blocked with 3% bovine serum albumin (BSA) in TBS-tween (19 mM Tris, 137 mM NaCl, 2.7 mM KCl, and 0.1% Tween20). Finally, the membranes were incubated with different primary and secondary antibodies (see the “Antibodies and chemicals” methods for details), and visualized with SuperSignal West Pico PLUS (Bio-Rad), a chemiluminescent horseradish peroxidase substrate. Luminescence signals were obtained on a VWR® Imager CHEMI Premium gel analysis system and quantified using FIJI(ImageJ) (NIH, USA).

### 2.8 SynGO analysis

GO analysis for cellular compartment (CC) was performed using SynGO v.11.2 (20), using proteins annotated as official gene symbols.

### 2.9 Analysis of differentially expressed proteins in the brain sheddome after GM6001 treatment

Four 10-week-old female C57BL6J mice were treated either with GM6001 or vehicle control. GM6001 (Bio-Techne R&D Systems) was administered intraperitoneally at 100 mg/kg. GM6001 was first dissolved in dimethyl sulfoxide (DMSO) and then in saline, with a final concentration of 10% DMSO in saline. Animals treated with the vehicle received injections of 10% DMSO in saline. Four hours after the administration, mice were sacrificed by cervical dislocation, and the cortices were extracted. Then the samples were processed as mentioned in the “Separation of membrane and soluble fractions” section, using 2 h and 100,000 *g* as the final ultracentrifugation parameters. The soluble samples were analyzed by LC-MS/MS. The raw data files were analyzed using FragPipe to obtain protein identifications and their respective label-free quantification values. Contaminant proteins were filtered out and proteins that were not identified/quantified consistently in same condition were removed as well. The MaxLFQ intensity values were converted to log2 scale, samples were grouped by conditions, and missing values were imputed using the “Missing not At Random” (MNAR) method. Protein-wise linear models combined with empirical Bayes statistics were used for the differential expression analyses. The limma package from R Bioconductor was used to generate a list of differentially expressed proteins for each pairwise comparison.

## 3 Results

**Figure 1:**
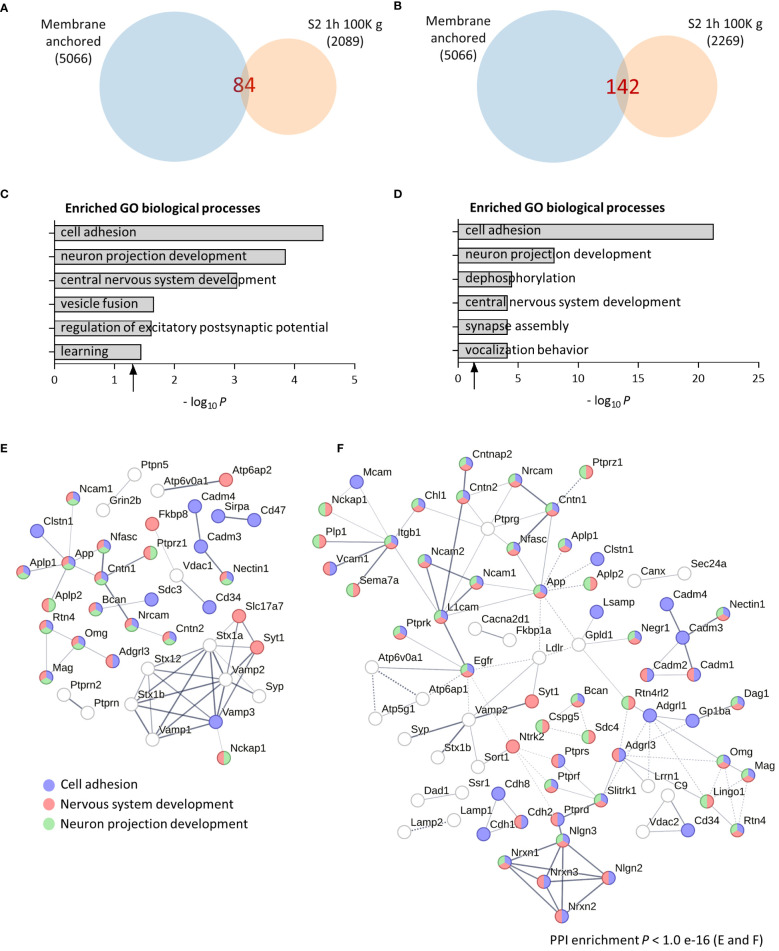
Computational analysis of the brain soluble fraction sheddomes. **(A)** Venn diagram of proteins from total soluble fraction S2 centrifuged for 1 h at 100,000 *g* (100K *g*) (2,089 identified proteins) and its membrane anchored subset (84 identified proteins). **(B)** Venn diagram of proteins from soluble fraction from “A” separated electrophoretically and analyzed in five independent fractions (2,269 identified proteins), showing that 142 could potentially undergo ES. **(C)** GO analysis of the total soluble fraction sheddome. **(D)** GO analysis of the soluble fraction sheddome separated electrophoretically in five fractions. **(E)** PPI network of the 84 proteins in the total soluble fraction sheddome highlighting the most significant biological processes (blue, cell adhesion; red, nervous system development; and green, neuron projection development). **(F)** PPI network of the 142 proteins in the total fraction sheddome separated into five fractions highlighting the most significant biological processes (blue, cell adhesion; red, nervous system development; and green, neuron projection development).

To evaluate the molecular and functional composition of the brain sheddome present in cortical soluble fractions, we first ultracentrifuged the cortex of an adult female mouse in TS buffer containing no detergents (see “Methods” for details) at 100,000 *g* (100K *g*) for 1 h. We obtained a list of 84 proteins in the soluble fraction sheddome (**Supplementary Table 2**) after bioinformatically selecting proteins containing either a transmembrane domain or a GPI anchor according to UniProt (**Figure 1A**). To evaluate agreement to previous bioinformatic analyses of brain sheddomes, we conducted GO analysis. This analysis revealed that the top six most enriched biological processes were cell adhesion, neuron projection development, CNS development, vesicle fusion, regulation of excitatory postsynaptic potential, and learning (**Figure 1C**, **Supplementary Table 2**). To increase the number of proteins detected in the soluble fraction sheddome, we loaded the same sample in an acrylamide/bisacrylamide gel, separated the proteins electrophoretically, and cut out the acrylamide lane in five fractions (**Supplementary Figure 1**) to analyze the five fractions separately by LC-MS/MS. We excluded proteins with molecular weights below 40 kDa to focus on the most prominent categories of shed proteins, such as cell adhesion molecules, receptors, and other membrane proteins, which generally have higher molecular weights. It is important to note, however, that ES can also occur in proteins of smaller sizes. This led to a 170% increase in the detected proteins in the sheddome, identifying a total of 142 proteins (**Figure 1B**, **Supplementary Table 3**). GO analysis showed again an enrichment for proteins regulating cell adhesion, neuron projection development, and CNS development (**Supplementary Table 3**). However, new pathways were detected such as dephosphorylation, synapse assembly, and vocalization behavior (**Figure 1D**). We performed a medium confidence PPI network analysis for both sheddomes observing that the PPI enrichment value was statistically significant for both datasets, indicating that the proteins in the networks are biologically connected. We highlighted those proteins involved in cell adhesion (blue), neuron projection development (green), and CNS development (red) based on our computational analysis. The depicted PPI networks include several proteins known to undergo ES, including Neural cell adhesion molecule (NCAM), Neuronal cell adhesion molecule (NRCAM), Amyloid-beta precursor protein (APP), Contactin-associated protein-like 2 (CNTNAP2), Neurexins 1 to 3 (NRXN1, 2, and 3), Neuroligin 2 (NLGN2), and various members of the protein Tyrosine phosphatase family (PTP) (**Figures 1E, F**). Taken together, these data indicate that the functional composition of the brain soluble fraction sheddome is similar to that of previously described sheddomes, involving processes like cell adhesion, axonal regulation, synapse assembly, and CNS development (12).

**Figure 2:**
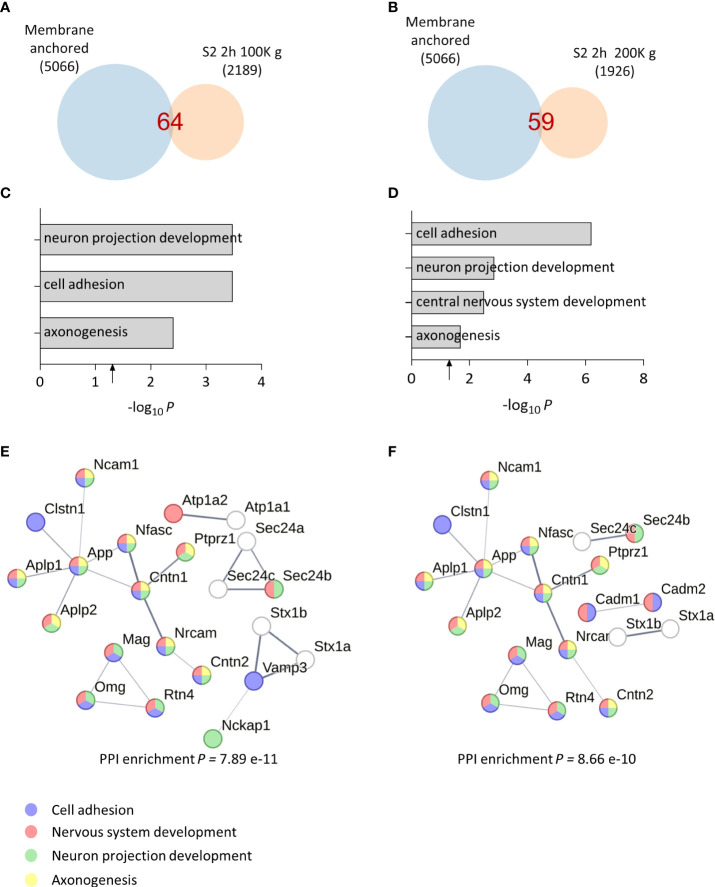
Bioinformatic analysis of optimized soluble fraction sheddome. **(A)** Venn diagram of proteins from total soluble fraction S2 centrifuged for 2 h at 100K *g* (2,189 identified proteins), depicting its membrane anchored subset (64 identified proteins). **(B)** Venn diagram of proteins from total soluble fraction S2 centrifuged 2 h at 200K *g* (1,926 identified proteins), showing that 59 could undergo ES. **(C)** GO analysis of the soluble fraction sheddome centrifuged for 2 h at 100K g. **(D)** GO analysis of the soluble fraction sheddome centrifuged for 2 h at 200K g. **(E)** PPI network of the 64 proteins in the soluble fraction sheddome centrifuged for 2 h at 100K *g* highlighting the most significant biological processes (blue, cell adhesion; red, nervous system development; green, neuron projection development, and yellow, axonogenesis). **(F)** PPI network of the 59 proteins in the soluble fraction sheddome centrifuged for 2 h at 200K *g* highlighting the most significant biological processes (blue, cell adhesion; red, nervous system development; green, neuron projection development; and yellow, axonogenesis).

Upon detecting synaptic vesicle proteins in our sheddome samples, we opted to extend the centrifugation duration and intensify the speed to guarantee the removal of vesicles. This adjustment aims to minimize any potential contamination with full-length proteins, which could interfere with our analysis. We homogenized cortices from two female mice in TS buffer, divided the S1 homogenate in two fractions, and centrifuged them for 2 h at 100K *g* or 200K *g*. Venn diagrams for the 100K *g* and 200K *g* samples show that the number of detected proteins in the sheddomes were similar, 64 and 59 proteins, respectively (**Figures 2A, B** and **Supplementary Table 4**). This number was 24% and 30% lower than the sample centrifuged for 1 h at 100K *g*, indicating that increasing the centrifugation time to 2 h may help eliminate undesired proteins. GO analysis revealed an enrichment for proteins regulating cell adhesion, neuron projection development, CNS development, and axonogenesis (**Figures 2C, D**, **Supplementary Table 4**), being very similar to processes enriched in samples centrifuged for 1 h (**Figures 1C, D**). We again performed a PPI network highlighting proteins involved in cell adhesion (blue), neuron projection development (green), CNS development (red), and axonogenesis (yellow), based on our computational analysis (**Figures 2E, F**). We also performed GO analysis for the P2 2 h 100K *g* sample showing totally different enriched biological processes, such as intracellular protein transport (red) and regulation of protein localization to plasma membrane (blue) (**Supplementary Figure 2A**, **Supplementary Table 5**). Finally, we performed a PPI network with the P2 fraction to visualize the proteins contained in this sample and their biological interactions (**Supplementary Figure 2B**, **Supplementary Table 5**). In P2, we found proteins regulating synaptic vesicle exocytosis (BIN1, AP2A1, ATP8A1, etc.), the actin cytoskeleton (CAMK2B or ACTB), or proteins regulating cytosolic calcium levels (ATP2B2, SLC8A1, or MYO5A), to name a few. This shows the expected differences between the S2 and the P2 samples, supporting an adequate subcellular fractionation of the tissue.

**Figure 3:**
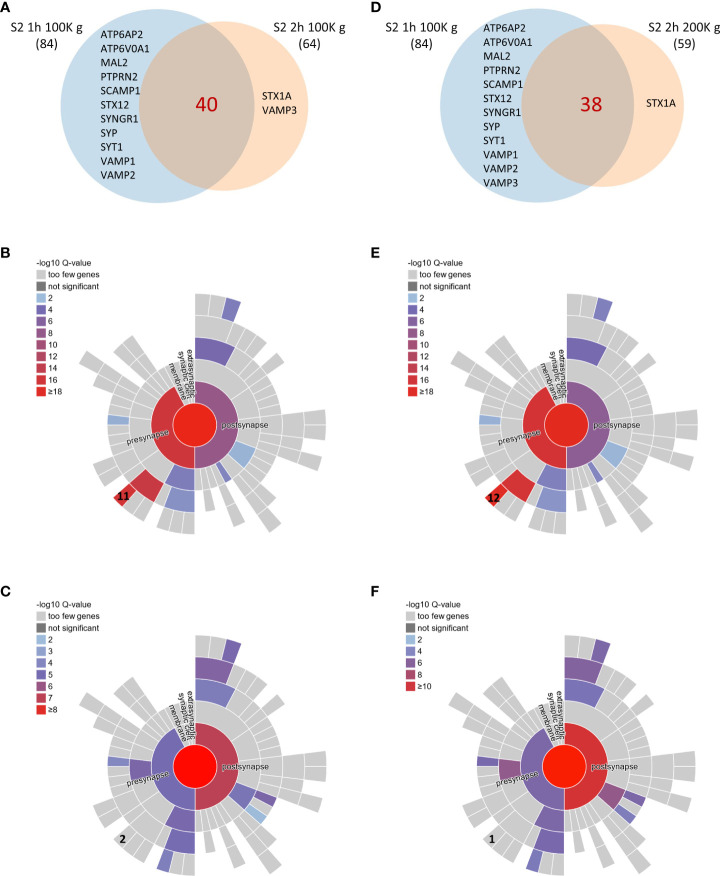
Comparison of brain soluble fraction sheddomes obtained at different ultracentrifugation conditions. **(A)** Venn diagram of shed proteins from soluble fraction centrifuged 1 h at 100,000 *g* (S2 1 h 100K *g*) (84 identified proteins) and from soluble fraction centrifuged 2 h at 100,000 *g* (S2 2 h 100K *g*) (64 identified proteins). **(B)** Sunburst plot for cellular compartment of the 44 proteins present in S2 1 h 100K *g* sheddome only, showing that “integral component of synaptic vesicle membrane” is significantly represented with 11 proteins expressed in that CC out of 44. **(C)** Sunburst plot for cellular compartment of the 64 proteins present in S2 2 h 100K *g* sheddome, showing that “integral component of synaptic vesicle membrane” is no longer significantly enriched with only 2 proteins expressed in that CC, out of the 64. **(D)** Venn diagram of shed proteins from soluble fraction centrifuged 1 h at 100K *g* (S2 1 h 100K *g*) (84 identified proteins) and from soluble fraction centrifuged 2 h at 200K *g* (S2 2 h 200K *g*) (64 identified proteins). **(E)** Sunburst plot for cellular compartment of the 38 proteins present in S2 1 h 100K *g* sheddome only, showing that “integral component of synaptic vesicle membrane” is significantly represented with 12 proteins expressed in that CC out of 38. **(F)** Sunburst plot for cellular compartment of the 59 proteins present in S2 2 h 100K *g* sheddome, showing that “integral component of synaptic vesicle membrane” is no longer significantly enriched with only 1 protein expressed in that CC out of the 59.

To further understand how increasing ultracentrifugation time and speed impacts the composition of the sheddome, we represented a Venn diagram with S2 1 h 100K *g* and S2 2 h 100K *g* samples (**Figure 3A**). We found an overlap of 40 proteins between the samples (**Supplementary Table 6**). We then decided to employ SynGO, a tool that systematically annotates synaptic genes using sunburst plots. In this type of graph, inner rings represent parent terms of more specific child terms in the outer rings, color-coded according to enrichment *Q*-value. Sunburst plots depicting CCs show that from the 34 proteins that were found only in S2 1 h 100K *g*, 11 are classified as integral components of synaptic vesicle membranes, including Vesicle-associated membrane protein 1 (VAMP1), VAMP2, Synaptophysin (SYP), and Synaptotagmin 1 (SYT1) (**Figure 3B**). After increasing the centrifugation time to 2 h, we only found two proteins expressed in that CC, i.e., Sintaxin1 (STX1A) and VAMP3 (**Figure 3C**, **Supplementary Table 6**). We then performed a similar analysis between S2 1 h 100K *g* and S2 2 h 200K *g* (**Figure 3D**). The data we obtained show very similar results to those found comparing 1 h and 2 h centrifugation times at 100K *g*: 11 proteins known to be expressed in the membrane of synaptic vesicles were found only in the S2 1 h 100K *g* sample (**Figure 3E**, **Supplementary Table 6**), while only one was found after centrifuging for 2 h at 200K *g* (**Figure 3F**, **Supplementary Table 6**). Interestingly, SynGO analysis of proteins only found in sample S2 1 h 100K *g* (corresponding to the light blue section of the Venn diagram) showed an enrichment on the CC “integral component of synaptic vesicle membrane” (**Figures 3B, E**, 11 out of 44 and 12 out of 46 proteins, respectively). When analyzing all the proteins found in S2 2 h 100K *g*, that enrichment was lost (**Figures 3A, C**), as only two of the identified proteins were classified as belonging to that CC. Similar results were observed when comparing the proteins found in the S2 2 h 200K *g* sample: only one protein remained in that CC and the enrichment for “integral component of synaptic vesicle membrane” was again lost (**Figures 3D, F**). Altogether, these data indicate that increasing the centrifugation time from 1 h to 2 h might help eliminate vesicles from the S2 sample, while increasing the speed from 100K *g* to 200K *g* barely has any effect on the protein composition of the sample. Thus, we consider that the most convenient conditions for the extraction of the brain soluble fraction sheddome were 2 h at 100K *g*.

**Figure 4:**
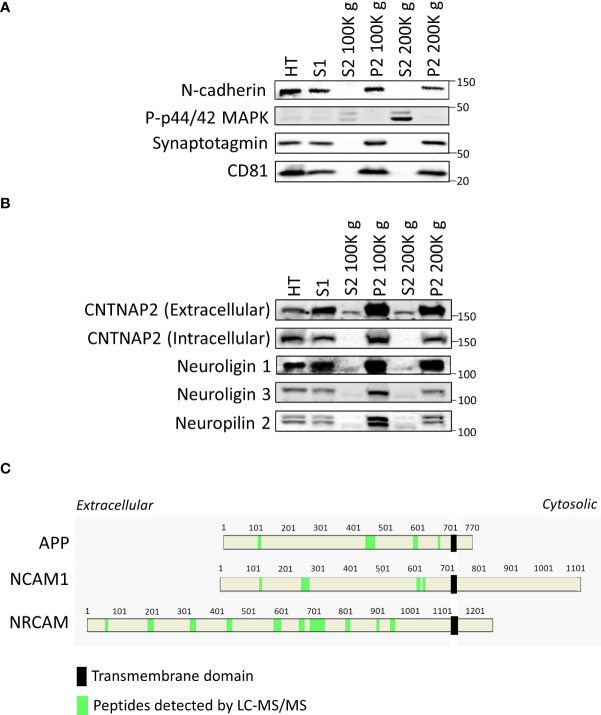
Validation of the protocol by Western blot. **(A)** Western blots of validation of tissue fractionation of mouse cortex, including the total homogenate (HT), S1, S2 centrifuged at 100K *g*, its P2 fraction, and the S2 centrifuged at 200K *g* and its P2 fraction using a membrane marker (N-cadherin), a cytosolic marker (p-p44/42 MAPK), a synaptic vesicle marker (synaptotagmin), and an extracellular vesicle marker (CD81). **(B)** Western blots demonstrate the presence of smaller shed ectodomains of CNTNAP2, Neuroligin 1 and 3, and Neuropilin 2 in S2 fractions. **(C)** Relative position of peptides detected by LC-MS/MS in green within the amino acid sequence of APP, NCAM1, and NRCAM in the S2 2 h 100K *g* MS sample, highlighting the transmembrane domain with a black rectangle.

After conducting proteomic and bioinformatic analyses of the samples, we opted to validate our methodology by Western blotting. As anticipated, we observed a signal using the antibody against the membrane protein N-cadherin on the total homogenate (HT), the S1 fraction, and on both P2 fractions (**Figure 4A**), all of which contain cell membranes. We also found an enrichment on the signal with the cytosolic phospho-p42/44 MAPK on the soluble fractions S2 100K *g* and 200K *g* (**Figure 4A**). Moreover, we observed a lack of signal with the synaptic vesicle marker Syt1 and with the extracellular vesicle marker CD81 in both S2 samples, corroborating the elimination of vesicles in the S2 samples (**Figure 4A**).

We proceeded to evaluate the presence of shed ectodomains in the S2 samples. We first used antibodies against the extracellular and the intracellular regions of CNTNAP2, a protein known to undergo ES (12). The extracellular directed antibody detected a band in the S2 samples, with a molecular weight just below that of the full-length protein (**Figure 4B**). To corroborate that the protein we detected in the S2 samples corresponded to shed CNTNAP2, we employed an antibody recognizing the intracellular portion of CNTNAP2, with which we did not observe any signal in the S2 samples. However, we detected a band in the P2 fractions (**Figure 4B**). These data indicate that the protein detected in the S2 fractions is the product of ES of CNTNAP2. We then decided to evaluate the shed ectodomains of three other proteins known to undergo ES, i.e., Neuroligin 1, Neuroligin 3, and Neuropilin 2 (8, 21, 22). As expected, we detected smaller molecular weight bands for all the mentioned proteins in the S2 fractions compared to those in the HT, S1, and P2 fractions (**Figure 4B**), supporting the idea of the presence of shed ectodomains in the S2 samples. All the original blots are presented in **Supplementary Figure 3**, with membrane identities and order of probing/reprobing/stripping of antibodies. Similarly, for three other proteins known to undergo ES, such as APP, NCAM1, and NRCAM, only peptides corresponding to the extracellular domains were detected by LC-MS/MS (in light green within the protein sequence, **Figure 4C**). This supports the idea that the detected proteins might be shed products of the full-length proteins, not originating from extracellular vesicles or cell debris, but rather from ES.

**Figure 5:**
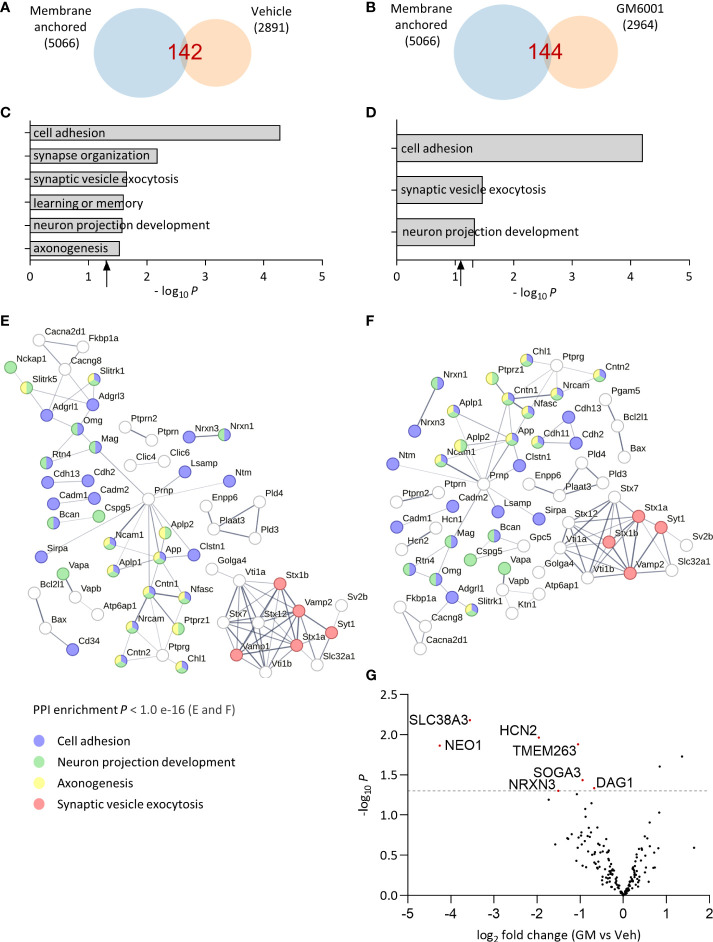
Effect of broad metalloprotease inhibition by GM6001 on the brain soluble fraction sheddome. **(A)** Venn diagram of proteins from total soluble fraction S2 centrifuged for 2 h at 100,000 *g* from vehicle (Veh)-treated female C57BL6J mice (*n* = 4, 2,089 identified proteins) and its membrane anchored subset (142 proteins, at least two occurrences). **(B)** Venn diagram of proteins from total soluble fraction S2 centrifuged for 2 h at 100,000 *g* from GM6001 (GM)-treated female C57BL6J mice (*n* = 4, 2,964 identified proteins) and its membrane anchored subset (144 proteins, at least two occurrences). **(C)** GO analysis of the total soluble fraction sheddome from vehicle-treated mice. **(D)** GO analysis of the soluble fraction sheddome from GM mice. **(E)** PPI network of the 142 proteins in the soluble fraction sheddome of vehicle-treated mice. **(F)** PPI network of the 144 proteins in the brain soluble fraction sheddome from GM-treated animals highlighted. For both **(E)** and **(F)**, the most significant biological processes have been highlighted (blue, cell adhesion; red, nervous system development; and green, neuron projection development). **(G)** Volcano plot of GM vs. Veh-treated mice sheddome proteins. The dashed line in the “*y*” axis corresponds to *y* = 1, 3 or *p* = 0.05. Proteins that reached statistical significance are indicated with their names and highlighted in red.

After identifying the optimal conditions for analyzing the brain soluble fraction sheddome (2 h, 100K *g*), we evaluated the effect of administering the broad-spectrum inhibitor of zinc-dependent proteases GM6001 (GM) on the composition of the brain soluble fraction sheddome. GM inhibits several members of the matrix metalloproteinases (MMPs) and the “A disintegrin and metalloproteinase” (ADAM) families by chelating the zinc ion at the active sites of the enzymes (23–25). We administered GM (100 mg/kg) or a vehicle control intraperitoneally to 10-week-old female C57BL6J mice (*n* = 4 mice per group). Four hours later, the cortices were dissected and processed to obtain the brain soluble fractions. These conditions have been successfully used to evaluate the inhibition of CNTNAP2 ES after GM treatment (12). Venn diagrams for the vehicle and GM groups showed that the number of identified proteins in the sheddomes was similar, 142 and 144 proteins, respectively (**Figures 5A, B**, **Supplementary Table 7**). According to what we expected, the number of identified proteins in the soluble fraction sheddome increased by approximately 2.5 times compared to previous experiments due to the increase in the number of experimental samples. GO analysis performed in the vehicle sheddome revealed an enrichment in proteins regulating processes such as cell adhesion, synapse organization, synaptic vesicle exocytosis, learning and memory, neuron projection development, and axonogenesis (**Figure 5C**), similarly to what has been previously described. Interestingly, treatment with GM decreased the number of pathways regulated by the proteins in the sheddome, as axonogenesis, synapse organization, and learning and memory were no longer enriched in the GM sheddome (**Figure 5D**). We then represented PPI networks with the proteins identified on the sheddomes of vehicle- and GM-treated animals (**Figures 5E, F** respectively). We highlighted those proteins involved in cell adhesion (blue), neuron projection development (green), axonogenesis (yellow), and synaptic vesicle exocytosis (red) based on our GO analysis. The depicted PPI networks included proteins known to undergo ES such as Neurexins 1 and 3, CADM1 and 2, NRCAM, and APP. Moreover, interesting risk factors for neurodevelopmental disorders were newly identified in the brain soluble fraction sheddome such as Neurofascin (NFASC), Myelin-associated protein (MAG), or Latrophilin 3 (ADGLR3), among others. A volcano plot representing the quantification of proteins in the soluble fraction sheddome in GM *vs* vehicle groups (**Figure 5G**, **Supplementary Table 8**) showed a decrease in NRXN3. NRXN3 is shed by ADAM10 and 17 (14), proteases that are inhibited by GM at an IC_50_ of 8.1 and 7.5 nM, respectively (25), thus validating our approach. Neogenin1 (NEO1), also downregulated in the sheddome after GM treatment, is known to be regulated by intramembrane proteolysis (26), which is usually preceded by ES. This indicates that NEO1 might be regulated by metalloproteinase-dependent ES. Other proteins appeared to be decreased after treatment with GM, including Solute carrier family 38 member 3 (SLC38A3), Hyperpolarization activated cyclic nucleotide gated potassium and sodium channel 2 (HCN2), Transmembrane protein 263 (TMEM263), Suppressor of glucose, autophagy associated 3 (SOGA3), and Dystroglycan1 (DAG1). Of the 169 proteins that were quantified globally in this experiment, 7 were significantly decreased by GM, accounting for 4.1% of the total proteins. Altogether, these data demonstrate that inhibition of sheddases by GM6001 decreases ES of membrane proteins, further validating our model for evaluating sheddomes by extracting brain soluble fractions.

**Figure 6:**
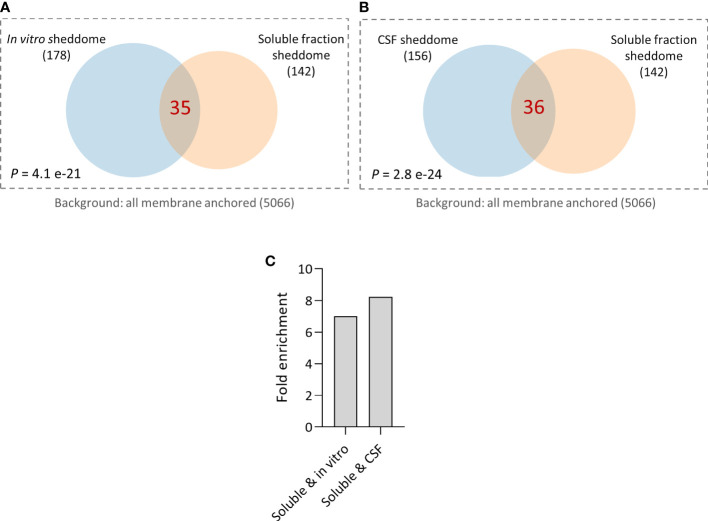
Comparison of published sheddomes and soluble fraction sheddome. **(A)** Venn diagram and hypergeometric testing shows a significant overlap of 35 proteins between the *in vitro* sheddome (178 identified proteins) and the soluble fraction sheddome (*n* = 4, 142 proteins). **(B)** Venn diagram and hypergeometric testing shows a significant overlap of 36 proteins between the sheddome *in vitro* (156 proteins) and the soluble fraction sheddome (*n* = 4, 142 proteins). **(C)** Fold enrichment of the overlap between the soluble and the *in vitro* sheddomes and between the soluble and the CSF sheddomes.

Finally, we decided to compare the molecular composition of the soluble fraction sheddome from the control group of the previous experiment with the *in vitro* and the CSF sheddomes described elsewhere (12). Hypergeometric testing showed a significant overlap between *in vitro* and the soluble fraction sheddomes (**Figure 6A**, **Supplementary Table 8**). We also found a statistically significant overlap between the CSF and the soluble fraction sheddomes (**Figure 6B**, **Supplementary Table 8**). There was almost a sevenfold enrichment in the overlap between the *in vitro* and the soluble fraction sheddomes as compared to randomly selecting 33 proteins from the transmembrane and GPI-anchored protein list (**Figure 6C**), while the fold enrichment was more than eightfold when analyzing the soluble and the CSF sheddomes (**Figure 6C**). These data show that the *in vitro*, the CSF, and the soluble fraction sheddomes have a similar molecular composition. However, there is another fraction of newly detected proteins in the soluble fraction sheddome that were not identified before: 105 new proteins were detected in the soluble fraction sheddome when compared to the *in vitro* sheddome, while 106 were newly detected in the soluble fraction sheddome when compared to the CSF sheddome. Among the newly identified proteins, we found proteins such as Neural cell adhesion molecule 1 (NCAM1), Cell adhesion molecule 2 (CADM2), and members of the Tyrosine-protein phosphatase family (PTPN5 and PTPRN), among others (**Supplementary Table 8**). Altogether, these data demonstrate that the analysis of the soluble fraction sheddome may lead to the detection of interesting new proteins that undergo ES.

## 4 Discussion

In this study, we introduce a refined methodology for the comprehensive analysis of brain sheddomes, aiming to complement and address limitations of past approaches, which are based on the exploration of cell culture media or CSF samples. ES, an essential molecular process involving the proteolytic cleavage of membrane-bound proteins, results in the release of soluble extracellular fragments with potential signaling functions (13). ES plays crucial roles in various aspects of CNS function and pathology, ranging from brain development to neurodegeneration (10–12). Consequently, robust methods to study ES are crucial for neuroscience, with potential applications in other research fields, including cancer, sepsis, renal, respiratory, and cardiovascular disease, among others (9, 27–30).

### 4.1 Current methodologies

Current methodologies for globally studying ES include the analysis of both conditioned media *in vitro* and CSF samples. These approaches offer several advantages. *In vitro* systems allow for a wide range of manipulations in a straightforward manner, facilitating the direction of research before progressing into more complex stages that require the use of research animals or human samples. CSF analysis can be used for biomarker discovery, allows for longitudinal studies in humans, and can provide real-time insights into processes occurring within the brain. It potentially enables the monitoring of CSF from the same patients over time, providing insights into how a specific disease affects the brain sheddome’s composition over time or how it is impacted by therapeutic interventions.

The study of conditioned media has been used to assess the secretome of neurons during apoptosis (31), breast carcinoma cells overexpressing the sheddase MT1–matrix metalloproteinase (32), or human epithelial cells (33). However, the use of *in vitro* secretomes is limited by the high abundance of albumin in the samples and contamination with proteins from dying cells. To circumvent these limitations, the Lichtenthaler group developed a technique called “secretome protein enrichment with click sugars (SPECS)” (34) and, more recently, an optimized version of the previous one, high-performance SPECS (hiSPECS) (17). Utilizing SPECS and hiSPECS enables the identification of *in vitro* and *ex vivo* secretomes (which includes both secreted and shed proteins) from cultured cells and brain slices. The Lichtenthaler group very elegantly and rigorously combines the use of SPECS or hiSPECS *in vitro* or *ex vivo* with advanced proteomics analysis of CSF, which has led to the identification of the substrates of several proteases including ADAM10 or BACE1, offering insight into therapeutic interventions (4, 18, 35, 36). Unfortunately, both SPECS and hiSPECS employ complex sample preparation and resources, making them less accessible for laboratories with limited resources. Moreover, secretomes include both secreted and shed proteins, thus hindering the specific identification of the molecular and functional properties of brain sheddomes.

The analysis of the *in vitro* and CSF proteomes also has other inherent limitations. The study of the sheddome *in vitro*, conducted in cultured cells, faces challenges in accurately representing disease and developmental mechanisms, often oversimplifying neuroinflammatory responses. Conversely, CSF sheddome analysis depends on shed proteins being able to diffuse away from the brain parenchyma. Moreover, its composition is influenced by its partial derivation from blood plasma and of debris coming from the natural cell turnover within the brain. On the other hand, there are also constraints on extractions in younger animals due to size limitations. Finally, the limited volume of CSF, when extracted from animals, complicates comprehensive analyses using various techniques or requires the use of increased number of animals.

### 4.2 Innovation of our approach and key findings

To circumvent some of these challenges, our group applied bioinformatics to SPECS-derived secretomes and human CSF samples, aiming to specifically identify shed proteins within the *in vitro* and the CSF sheddomes (12), separating them from secreted soluble proteins. In the current study, we introduce the use of brain soluble fractions, proteomics, and our bioinformatic filtering method to study the brain sheddome. This methodology is based on the activity of a type of proteases, referred to as sheddases, which lead to the release of the ectodomain of membrane proteins into the extracellular compartment. Thus, by extracting the soluble portion of a tissue, we can detect solubilized shed ectodomains, after properly eliminating cell membranes from the samples. To identify proteins undergoing ES within the soluble fraction proteome detected by LC-MS/MS, we bioinformatically select proteins that present at least one transmembrane domain or a GPI anchor, according to UniProt. Our methodology yields a substantial volume of sample from each animal, allowing for the use of various analytical techniques including LC-MS/MS, Western blot, and ELISA, thus contributing to the application of the 3R principles in animal research. This approach could also be applied to human biopsies or postmortem tissue.

Our study demonstrates the efficacy of this methodology, involving biochemical tissue separation to extract soluble fractions, mass spectrometry and bioinformatic analysis for the evaluation of the brain sheddome. We optimized the ultracentrifugation parameters by extending the ultracentrifugation time to 2 h, which enhanced the specificity of the sheddome composition, by significantly reducing proteins expressed in synaptic vesicles. This adjustment is crucial for reducing potential contamination with full-length proteins, which would otherwise interfere with our analysis. On the other hand, increasing the speed from 100K *g* to 200K *g* had little to no effect on the sheddome composition, as evidenced by a lack of effect on the number of proteins in the sheddome classified as “integral components of synaptic vesicle membrane” according to SynGO. While previous studies aimed at studying ES of specific proteins employed ultracentrifugation parameters of 1 h and 100K *g* (8, 12), suitable for detecting ES of particular proteins, these conditions proved to be suboptimal for the global analysis of the brain sheddome. Inadequate elimination of vesicles from the sample may impact the bioinformatic evaluation of the sheddomes. However, this may not significantly impact the study of specific proteins using alternative techniques such as Western blot, provided that appropriate controls are employed, including the use of antibodies targeting the intracellular domains of the protein of interest.

Additionally, we show that fractionating the sample after electrophoretically separating the proteins in an acrylamide gel and excluding smaller proteins significantly enhances the number of proteins identified in the brain soluble sheddome. This increase in the number of detected proteins within the brain sheddome and, thus, the statistically significant biological processes enriched in the sample may provide a deeper understanding of the processes governed by ES. It is important to note that this approach may not be feasible for laboratories with limited funding, given the associated increase in proteomics analysis costs.

For an adequate validation of our methodology, we increased the number of animals and administered them with GM6001, a broad-spectrum protease inhibitor, to study the impact of protease inhibition on the brain soluble fraction sheddome. Interestingly, 4% of the proteins were significantly decreased after GM treatment, supporting the potential of our methodology to evaluate shed proteins. Strikingly, the number of significant pathways regulated by shed proteins in the sheddome of the GM group was reduced from six in the vehicle control to three despite the abundance of identified proteins being very similar in both. The pathways not regulated by the GM sheddome were synapse organization, learning and memory, and axonogenesis. The application of a broad-spectrum protease inhibitor indicates how changes in sheddase activity could alter pathways critical for neurological and neuropsychiatric functions, underscoring the biological importance of our findings. As a final step of the validation of our protocol, we performed comparative analyses between the soluble fraction sheddome and the published *in vitro* and the CSF sheddomes, revealing significant overlaps, which supports the molecular similarity among these sheddomes.

The proteins identified in the brain soluble fraction sheddome align with known sheddome molecular and functional components, emphasizing the involvement of shed ectodomains in critical biological processes, such as cell adhesion, neuron projection development, and axonogenesis. PPI network analysis further elucidates the interconnectedness of proteins participating in ES. Notably, the detection of newly identified proteins in the soluble fraction sheddome suggests that this methodology may offer a novel approach for uncovering proteins undergoing ES, warranting further investigation into their roles and relevance for health and disease.

### 4.3 Implications and future directions

The use of this technique for the study of ES has the potential to enhance our understanding of the role of ES in health and disease. It may also open avenues for identifying new therapeutic targets and biomarkers, possibilities that should be explored through subsequent research. By identifying proteins regulated by ES, this technique opens avenues for future studies to explore how the shedding of specific proteins may impact brain function, as earlier done in other proteomics analysis (12). Applying this methodology to disease-specific mouse models or human samples, and integrating it with other omics approaches, represents a potential strategy for achieving a more comprehensive understanding of brain pathophysiology, pending further research. The utilization of human brain samples for studying the brain sheddome will necessitate rigorous tissue management practices to avert degradation. This caution is particularly vital for samples obtained postmortem, as any degradation can significantly alter the sheddome’s composition, skewing analytical outcomes and interpretations.

## 5 Conclusion

In summary, this study introduces a methodology for analyzing brain sheddomes, overcoming some of the limitations of conventional approaches, and offering a complementary tool to add to the available methods. This methodology presents several advantages: (i) it identifies shed proteins; (ii) it generates ample samples that can be analyzed by several techniques on the same specimen; (iii) it might help reduce the number of animals needed to perform research; (iv) the technique can be applied to mice at any postnatal age; and (v) the employed resources are accessible to many research laboratories. The optimized ultracentrifugation parameters and validated global assessment of ES may contribute to a solid foundation for future research. Exploring the extension of this methodology beyond neuroscience, including fields such as cancer and cardiovascular research, presents an intriguing prospect that warrants further exploration, offering a valuable tool for unraveling ES and its impact in health and disease.

## Data availability statement

The original contributions presented in the study are included in the article/**Supplementary Material**, further inquiries can be directed to the corresponding author.

## Ethics statement

The animal study was approved by the Ethical Committee of Universidad Complutense de Madrid (ref. PROEX 305.6/22). The study was conducted in accordance with the local legislation and institutional requirements.

## Author contributions

ML: Writing – review & editing, Writing – original draft, Investigation, Formal analysis, Data curation. TS: Writing – review & editing, Investigation, Formal analysis, Data curation. SIB: Writing – review & editing, Formal analysis, Data curation. SS: Writing – review & editing, Formal analysis. MOG: Writing – review & editing, Formal analysis. MDMS: Writing – review & editing, Writing – original draft, Visualization, Validation, Supervision, Software, Resources, Project administration, Methodology, Investigation, Funding acquisition, Formal analysis, Data curation, Conceptualization.

## Supplementary material

Supplementary Figure 1 Coomassie stained gel of electrophoretically separated proteins from the S2 1 h 100,000 *g* soluble fraction showing the five fractions cut out from the gel to be analyzed separately by LC-MS/MS.

Supplementary Figure 2 Computational analysis of membrane fraction P2 2 h 100K g**. (A)** GO analysis of the P2 2 h 100K *g* sheddome. **(B)** PPI network of the 315 proteins in the P2 2 h 100K *g* highlighting the two enriched biological processes in the sample (red, intracellular protein transport, and blue, positive regulation of protein localization to plasma membrane).

Supplementary Figure 3 Original blots corresponding to **Figure 4** with membrane identities and order of probing/reprobing/stripping of antibodies.

Supplementary Table 1 List of transmembrane and GPI-anchored proteins used for filtering proteins belonging to the soluble fraction sheddomes and used as background for GO analysis in DAVID.

Supplementary Table 2 List of proteins in the soluble fraction sheddome S2 1 h 100K *g* (including entrez, official gene symbol and gene names) and its GO analysis showing the significantly enriched biological processes.

Supplementary Table 3 List of proteins in the soluble fraction sheddome S2 1 h 100K *g* electrophoretically separated in five fractions (including entrez, official gene symbol, and gene names) and its GO analysis showing the significantly enriched biological processes.

Supplementary Table 4 List of proteins in the soluble fraction sheddomes S2 2 h 100K *g* and S2 2 h 200K *g* (including entrez, official gene symbol, and gene names) and their GO analysis showing the significantly enriched biological processes.

Supplementary Table 5 List of proteins in the membrane fraction P2 2 h 100K *g* and its GO analysis showing the significantly enriched biological processes.

Supplementary Table 6 Comparison of proteins in soluble sheddomes S2 1 h 100K *g* and S2 2 h 200K *g* and comparison of sheddomes S2 1 h 100K *g* and S2 2 h 200K *g*, including lists of proteins present only in one sample, on the other sample or in both. Bold letters are used to highlight proteins that belong to the cellular compartment “integral component of synaptic vesicle membrane.”

Supplementary Table 7 List of proteins, GO analysis, and quantitative data of vehicle- and GM6001-treated mice brain soluble fraction sheddomes.

Supplementary Table 8 Comparison and list of proteins in soluble fraction S2 2 h 100K *g* (*n* = 4) and *in vitro* sheddomes, and comparison and list of proteins of S2 2 h 100K *g* (*n* = 4) and CSF sheddomes.
