# Supplementary figures and images for: A methodology to globally assess ectodomain shedding using soluble fractions from the mouse brain
Source: Front Psychiatry. 2024 Jun 19;15:1367526. doi: 10.3389/fpsyt.2024.1367526 (PMC11219901; doi:10.3389/fpsyt.2024.1367526)

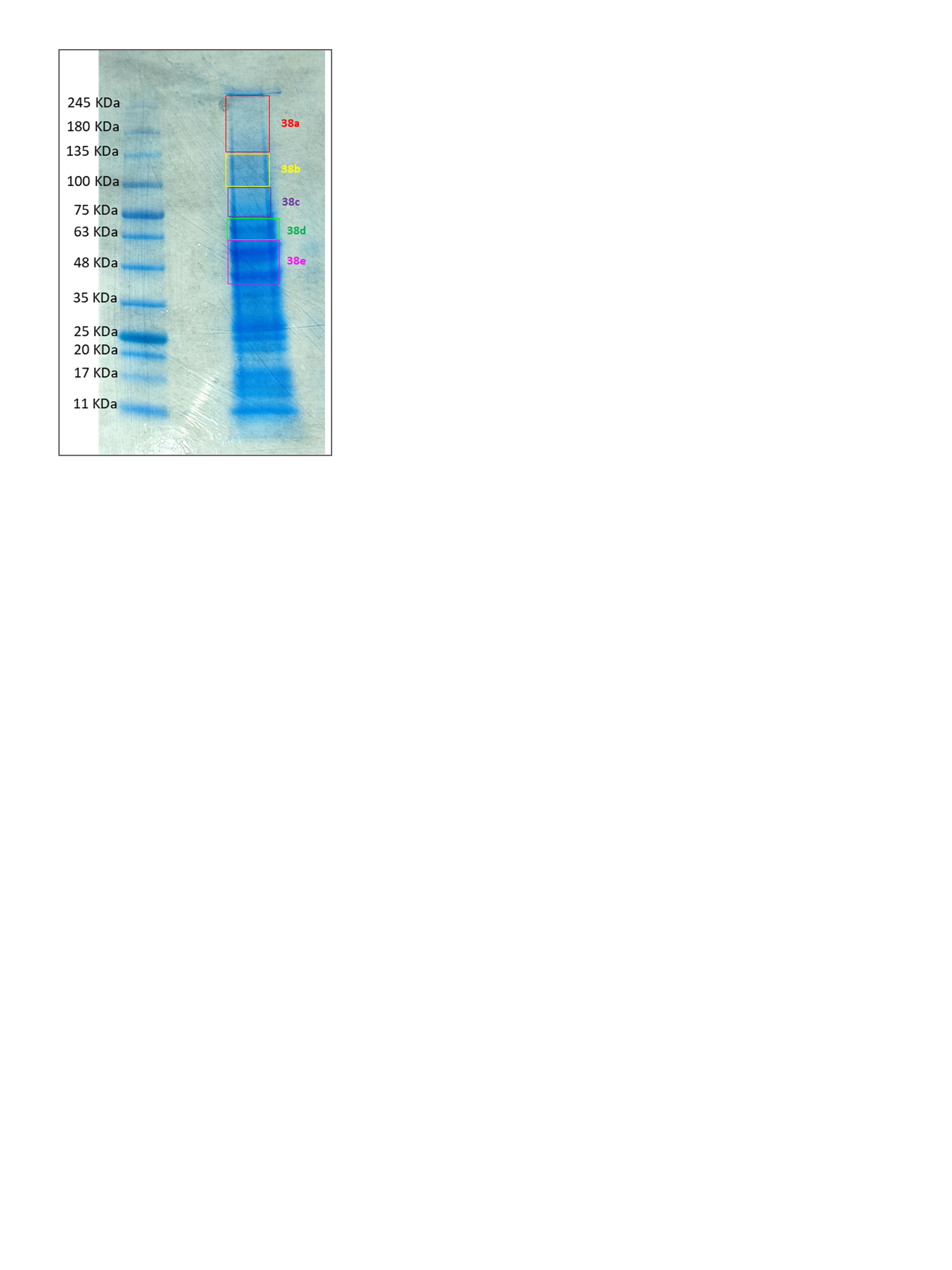

Supplement: Supplementary Figure 1 — Coomassie stained gel of electrophoretically separated proteins from the S2 1 h 100,000 g soluble fraction showing the five fractions cut out from the gel to be analyzed separately by LC-MS/MS. [file Image_1.tif]

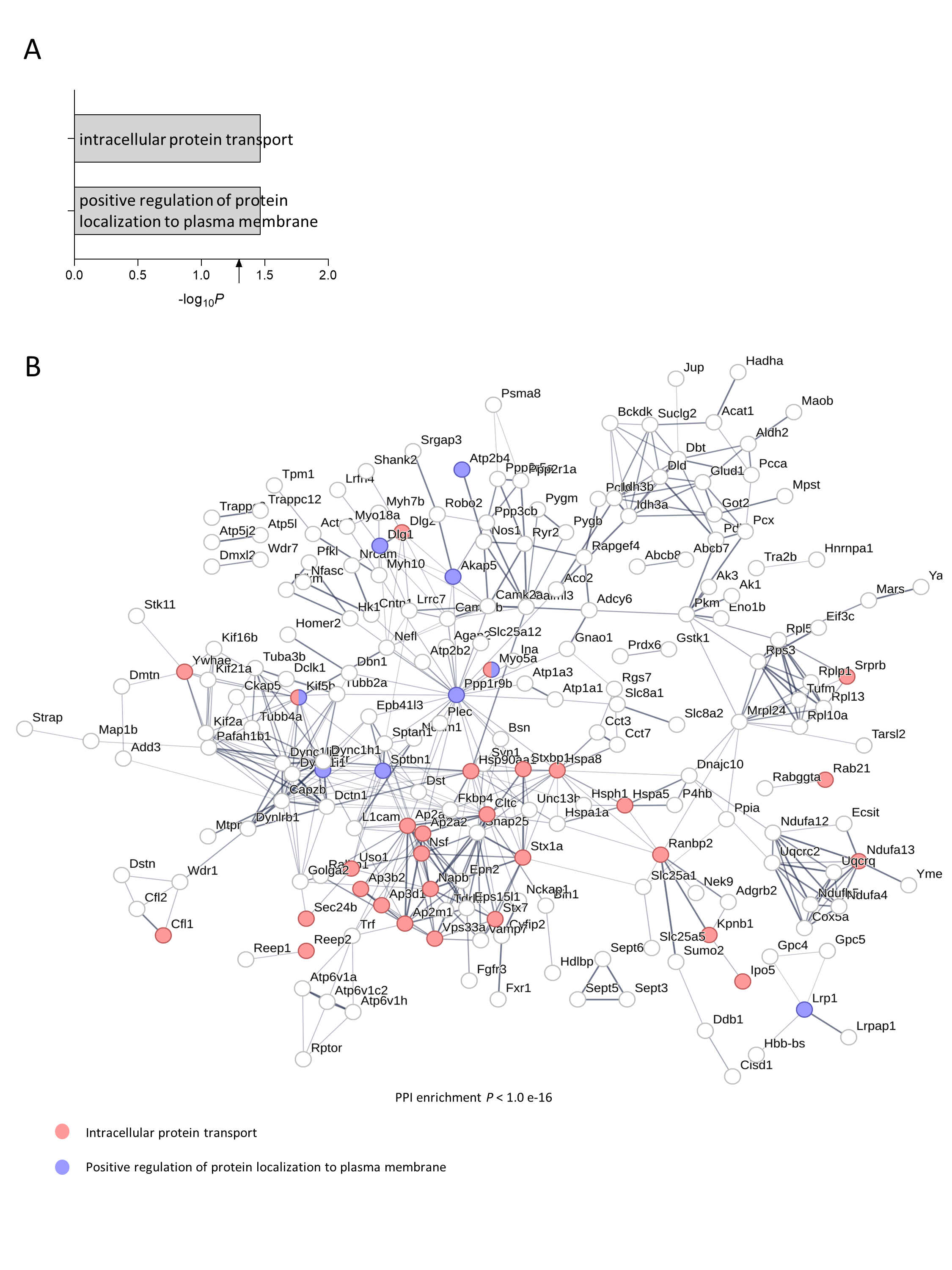

Supplement: Supplementary Figure 2 — Computational analysis of membrane fraction P2 2 h 100K g. (A) GO analysis of the P2 2 h 100K g sheddome. (B) PPI network of the 315 proteins in the P2 2 h 100K g highlighting the two enriched biological processes in the sample (red, intracellular protein transport, and blue, positive regulation of protein localization to plasma membrane). [file Image_2.tif]

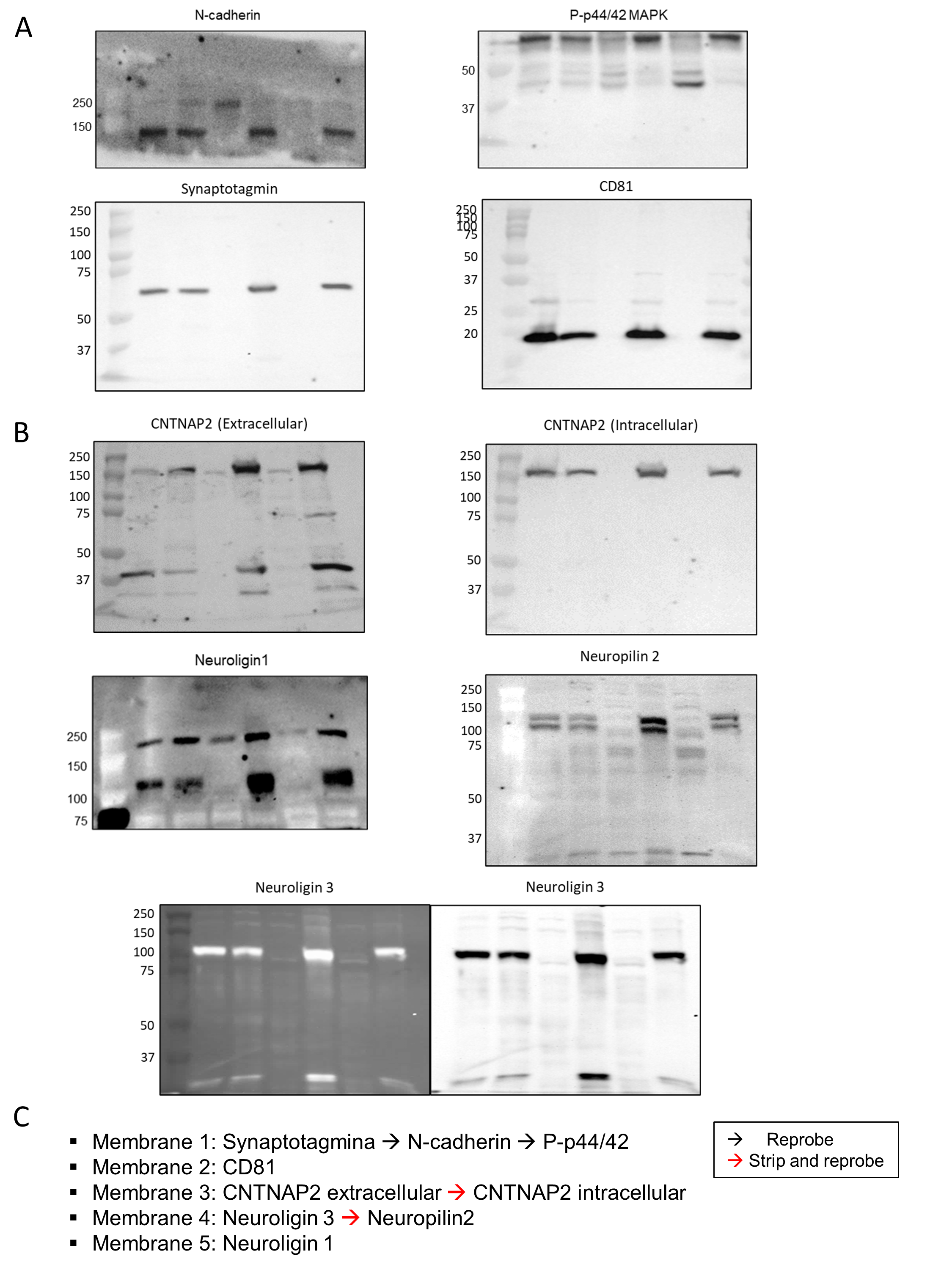

Supplement: Supplementary Figure 3 — Original blots corresponding to Figure 4 with membrane identities and order of probing/reprobing/stripping of antibodies. [file Image_3.tif]
